# Alterations in the Fecal Microbiota of Patients with HIV-1 Infection: An Observational Study in A Chinese Population

**DOI:** 10.1038/srep30673

**Published:** 2016-08-01

**Authors:** Zongxin Ling, Changzhong Jin, Tiansheng Xie, Yiwen Cheng, Lanjuan Li, Nanping Wu

**Affiliations:** 1 Collaborative Innovation Center for Diagnosis and Treatment of Infectious Diseases, State Key Laboratory for Diagnosis and Treatment of Infectious Diseases, the First Affiliated Hospital, School of Medicine, Zhejiang University, Hangzhou, Zhejiang, 310003, China

## Abstract

The available evidence suggests that alterations in gut microbiota may be tightly linked to the increase in microbial translocation and systemic inflammation in patients with human immunodeficiency virus 1 (HIV-1) infection. We profiled the fecal microbiota as a proxy of gut microbiota by parallel barcoded 454-pyrosequencing in 67 HIV-1-infected patients (32 receiving highly active antiretroviral therapy [HAART] and 35 HAART naïve) and 16 healthy controls from a Chinese population. We showed that α-diversity indices did not differ significantly between the healthy control and HIV-1-infected patients. The ratio of Firmicutes/Bacteroidetes increased significantly in HIV-1-infected patients. Several key bacterial phylotypes, including *Prevotella*, were prevalent in HIV-1-infected patients; whereas *Phascolarctobacterium*, *Clostridium* XIVb, *Dialister* and *Megamonas* were significantly correlated with systemic inflammatory cytokines. After short-term, effective HAART, the viral loads of HIV-1 were reduced; however, the diversity and composition of the fecal microbiota were not completely restored. and the dysbiosis remained among HIV-1-infected subjects undergoing HAART. Our detailed analysis demonstrated that dysbiosis of fecal microbiota might play an active role in HIV-1 infection. Thus, new insights may be provided into therapeutics that target the microbiota to attenuate the progression of HIV disease and to reduce the risk of gut-linked disease in HIV-1-infected patients.

HIV-1 infection is characterized by rapid and substantial CD4^+^ T-cell depletion and chronic immune system activation, with more than 33 million people affected worldwide and more than 2.5 million additional cases each year^1^. High prevalence of HIV-1 infection has been reported in recent years across all regions of China, especially in men who have sex with men (MSM)^2^. Based on accumulating evidence, HIV-1 infection is tightly linked to the gastrointestinal tract, which is a primary site of viral replication^3^,^4^. A significant depletion of CD4^+^ T-cells occurs in the intestinal mucosa, preferentially in the gut-associated lymphoid tissue (GALT) in the early phase of HIV-1 infection, which results in damage to and dysfunction of the gastrointestinal system^5^. The consequence of the deterioration of gut homeostasis is chronic immune activation because of the increased permeability of the gut, a dysregulated intestinal immune barrier and, ultimately, the translocation of bacterial products such as lipopolysaccharides (LPS), peptidoglycans and bacterial DNA into the circulatory system^6^,^7^,^8^. The aggressive cycle of immune activation in turn promotes viral replication and disease progression. Persistent immune activation is one of the strongest predictors of HIV disease progression and likely drives progression of the disease to acquired immune deficiency syndrome (AIDS). Highly active antiretroviral therapy (HAART) appears to at least partially restore gut integrity and reconstitute GALT CD4^+^ T-cells, which can help prevent HIV infection sequelae and dissemination^7^,^9^.

A normal gastrointestinal tract harbors a complex community of balanced microorganisms that functions in myriad ways that is beneficial to the host, which plays a fundamental role on the induction, training, and function of the host immune system^10^. Gori *et al*. firstly observes a high prevalence of *Pseudomonas aeruginosa* and *Candida albicans* as well as lower abundance of *Bifidobacteria* and *Lactobacilli* in the fecal microbiota compared with healthy controls, which indicates that potential intestinal dysbiosis exists in early HIV-1 infection^11^. The modulation of the mucosal immune system by altered gut microbiota overlaps with the salient features of HIV pathogenesis^12^ that are proposed to play a key role in HIV disease progression^13^. Additionally, in a previous study, the gut microbiota of HIV-positive individuals rarely resembles that of HIV-negative ones after long-term, successful HAART, the degree to which HAART restores health-associated prevalence varies across bacterial taxa^4^. The diversity of gut microbiota is directly correlated with the nutrient contents, and lower beneficial bacteria counts such as *Bifidobacteria* and *Lactobacilli* are found in the feces of HIV-infected subjects than in that of healthy controls^14^. These beneficial bacteria may interact with the GALT to preserve gut integrity, thereby decreasing the possible translocation of microbial products. Moreover, the gut microbiota represent a crucial line of resistance because microbial alteration of the gastrointestinal tract impairs colonization of the intestine by many pathogens using both direct and indirect (immune-mediated) mechanisms. In a recent study, dysbiosis of the gut microbiota was associated with the progression of HIV disease and tryptophan catabolism^13^. Supplementation of the diet with a prebiotic oligosaccharide mixture improved the composition of the gut microbiota, reduced the activation of sCD14 and CD4^+^ T-cells, and improved NK cell activity in HAART-naive HIV-infected individuals^15^. Additionally, specific gut microbiota are required to regulate the mucosal immune responses, and perturbations in the microbiota can result in a lack of immunoregulation, with the outgrowth of more pathogenic microbes and the promotion of inflammation. With the significant disruptions to mucosal immunity and the state of constant systemic immune activation well established for HIV infection, understanding the role of an altered gut microbiota in the sustainment of pathologic and chronic inflammation is of paramount importance.

However, most prior studies have been performed on samples obtained from HIV-1-infected patients in Western countries^4^,^9^,^13^,^16^,^17^,^18^,^19^,^20^,^21^,^22^,^23^,^24^,^25^, and little is known about the alterations of gut microbiota in HIV-1-infected Chinese population. As we all known, dietary habits are considered a major determinant contributing to the diversity of human gut microbiota^26^,^27^,^28^. The different dietary constitution between Chinese and Western population may result in disparities of the composition of gut microbiota. Because of the importance of the interactions between gut microbiota and mucosal immunity in patients with HIV infection, the aim of the present study was to identify differences in the fecal microbiota as a proxy of gut microbiota between healthy subjects and patients with HIV-1 infection in a Chinese population. Massively parallel barcoded 454-pyrosequencing that targeted the 16S rRNA gene V1-V3 hypervariable regions was used to identify the differences. With HAART-treated HIV patients included in the study, the results will help to elucidate the role of active HIV infection in shaping the composition of gut microbiota and in altering mucosal immunity. Thus, new insights may be provided into therapeutics that target the microbiota to attenuate the progression of HIV disease and to reduce the risk of gut-linked disease in people with HIV.

## Results

### Overall structural changes of HIV-associated fecal microbiota

**Figure 1:**
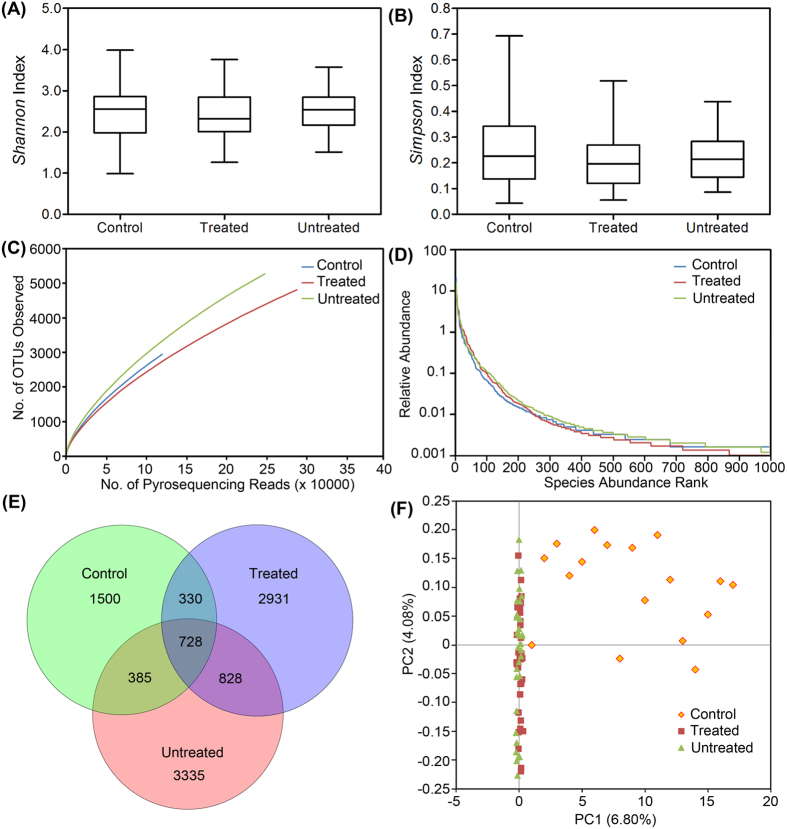
Comparison of fecal microbiota structure among healthy controls and treated and untreated HIV-1-infected groups. The Shannon (**A**) and Simpson (**B**) indices were used to estimate the diversity (i.e., a combined assessment of the number of 97% similar bacterial taxa and their abundance) of fecal microbiota. Rarefaction curves were used to estimate the richness (at a 97% level of similarity) of fecal microbiota among the three groups (**C**). The vertical axis shows the number of OTUs expected after sampling the number of tags or sequences shown on the horizontal axis. Rank abundance curves of bacterial OTUs derived from the three groups (**D**). The Venn diagram illustrates the overlap of OTUs in fecal microbiota among the three groups (**E**). Plot of principal coordinate analysis of the fecal microbiota based on the unweighted UniFrac metric (**F**).

From 83 samples, 654,246 high-quality sequences (62.1% of valid sequences (1,053,886,846 total reads) based on barcode- and primer-sequence filtering) with a median read length of 461 bp (range from 221 to 540 bp) were produced, with an average of 7,882 (range from 3,384 to 11,927) sequences per barcoded sample recovered for downstream analysis. A total of 119,360 sequences were obtained from healthy controls for phylogenetic analysis; whereas 534,886 sequences were obtained from patients with HIV (247,471 from 35 participants in the untreated group and 287,415 from 32 participants in the treated group). The total number of unique sequences from the 3 groups was 10,037, which represented all phylotypes. Specifically, 2,943 species-level OTUs in the healthy controls, 5,276 OTUs in the untreated group and 4,817 OTUs in the treated group were delineated at a 97% similarity level. The summary information is shown in Table 1, and detailed characteristics of each sample are shown in Table S1. Good’s coverage for the 3 groups was more than 98.0%, which indicated a great sequencing depth for the HIV-associated fecal microbiota. In the analysis of alpha diversity with Shannon and Simpson indices, the diversity of fecal microbiota in the patients with HIV, as a group, was similar to that of the healthy controls (Fig. 1A,B). Although the Shannon index was higher in patients with HIV and the Simpson index was lower than those indices in healthy controls, these differences were not statistically significant (P > 0.05). Based on the rarefaction analysis estimates, the trend in species richness in patients with HIV was also similar to that in the healthy controls (Figs 1C and S1). Based on analysis of the OTUs, a long tail in the rank abundance curves was observed, which indicated that the abundance of most OTUs was low (Fig. 1D). To better understand the shared richness among the three groups, a Venn diagram was developed to display the overlaps between groups. These diagrams showed that only 728 o OTUs of the total richness of 10,037 were shared among all samples, whereas 1,556 of 8,537 OTUs were shared between the samples of the HIV infected groups (Fig. 1E). Thus, more than twice the number of OTUs was detected in the HIV infected groups compared with that in the healthy controls. Beta diversity analysis determines the extent of the similarity between microbial communities by measuring the degree to which membership or structure is shared between communities. Despite significant inter-individual variation, the fecal microbiota from patients with HIV was separated clearly in the principal coordinates analysis from that of healthy controls (Fig. 1F). However, as shown in the unweighted UniFrac analysis (Figure S2), the HIV-treated and untreated groups were not separated into different clusters to any degree. Based on great depth of sequencing coverage in this study, the overall diversity of HIV-associated fecal microbiota was unaltered, although more OTUs and phylotypes were observed in patients with HIV.

### Associations between fecal microbiota and patients with HIV

A taxon-dependent analysis using the RDP classifier was conducted to describe the composition of HIV-associated fecal microbiota. Twelve phyla, which included Bacteroidetes, Firmicutes, Proteobacteria, Fusobacteria, Acidobacteria, Actinobacteria, Candidatus Saccharibacteria, Chloroflexi, Deinococcus-Thermus, Lentisphaerae, Synergistetes and Verrucomicrobia, were identified. The three most predominant phyla (Firmicutes, Bacteroidetes and Proteobacteria) in fecal samples accounted for >97% of the total sequences. Compared with healthy controls, the proportion of Firmicutes increased and the proportion of Bacteroidetes decreased in patients with HIV. From the sequences of fecal microbiota, a total of 209 genera were classified, with 123 genera in healthy controls and 191 in HIV patients. Of the total genera, 9 genera were predominant in healthy controls; whereas 15 genera were predominant in HIV patients. A predominant genus was defined as having >1% of the total DNA sequences, and these predominant genera accounted for 88.12% and 89.82% of the total sequences from healthy controls and patients with HIV, respectively. *Bacteroides*, *Prevotella* and *Faecalibacterium* are the three most predominant genera in both healthy controls and patients with HIV were *Bacteroides*, *Prevotella* and *Faecalibacterium*. In addition to the shared nine predominant genera in both groups, *Megamonas*, *Blautia*, *Parabacteroides*, *Veillonella*, *Parasutterella* and *Fusobacterium* were also abundant members of the fecal microbiota in patients with HIV. The correlations between the participants and the abundances of selected genera in the microbiota samples are shown in a heatmap (Figure S3), which shows the genus-level clustering according to the frequency within each sample. Consistent with the values for the alpha-diversity indices, such as the Shannon index, and with those for the beta-diversity metrics, such as the unweighted UniFrac analysis, clustering analysis of these genera highlighted similar distributions according to the health status of the host.

**Figure 2:**
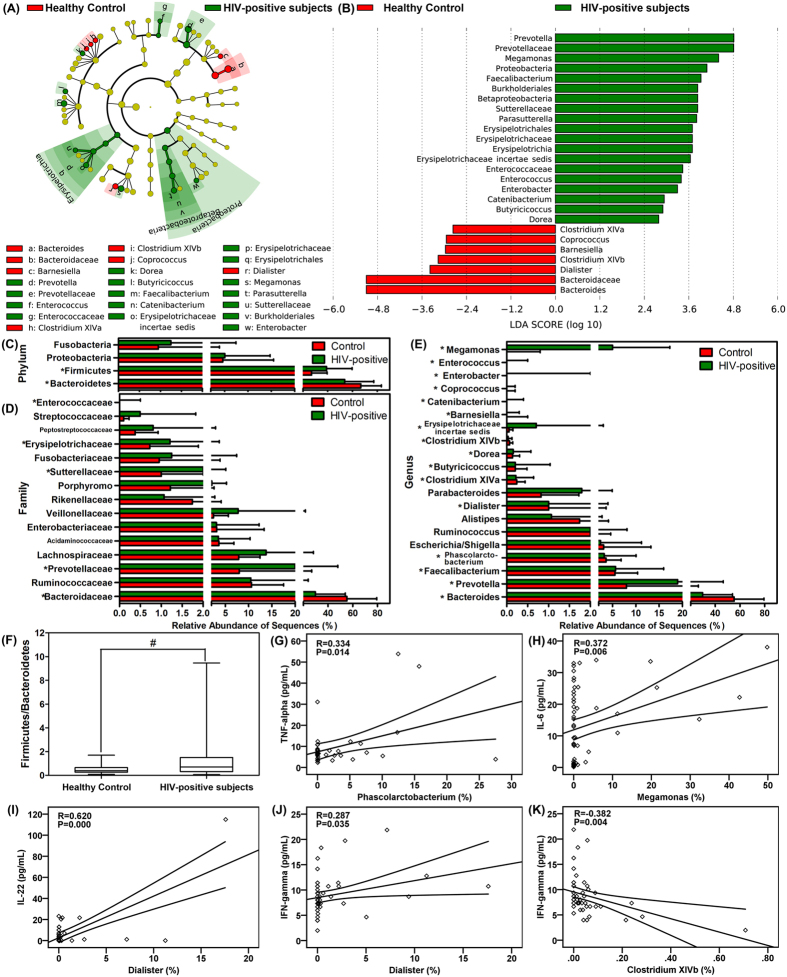
Taxonomic cladogram obtained using LEfSe analysis of the 16S sequences. LEfSe identifies the taxa with the greatest differences in abundance between healthy controls and HIV-1 positive individuals. (Red) healthy control-enriched taxa; (green) HIV-1-positive-enriched taxa. The brightness of each dot is proportional to the effect size (**A**). The HIV-1-positive-enriched taxa are indicated with a positive LDA score (green) and healthy control-enriched taxa have a negative score (red). Only the taxa with meeting a significant LDA threshold value of >2 are shown (**B**). Comparisons of the relative abundance at the level of bacterial phylum (**C**), family (**D**) and genus (**E**) in healthy controls and HIV-1 positive individuals; *P < 0.05; ^#^P < 0.01. The ratio of Firmicutes/Bacteroidetes in healthy controls and HIV-1 positive individuals (**F**). Correlations between TNF-α and the relative abundance of the genus *Phascolarctobacterium* (**G**), between IL-6 and the relative abundance of the genus of *Megamonas* (**H**), between IL-22 and the relative abundance of the genus *Dialister* (**I**), and between IFN-γ and the relative abundances of the genera *Dialister* (**J**) and *Clostridium* XIVb (**K**).

To identify the specific bacterial taxa associated with HIV, we compared the composition of the fecal microbiota of healthy controls and that of patients with HIV using the linear discriminant analysis effect size (LEfSe) method. A cladogram that represents the structure of the fecal microbiota and the predominant bacteria in the healthy control and HIV-positive patients is shown in Fig. 2A; with the largest differences in the taxa between the two communities displayed. The changes in the composition of the fecal microbiota in HIV-1-infected samples were also explored using the Mann-Whitney U-test at different taxon levels. The LEfSe analysis revealed 26 discriminative features (LDA score >2, Fig. 2B). Members of bacterial taxa in the Bacteroidetes were enriched in the healthy-control samples, whereas those in the Firmicutes and Proteobacteria were enriched in the HIV-positive patient samples, which could be used as biomarkers to discriminate the patients with an HIV infection. Despite high inter-individual variability, at the level of phyla, Firmicutes was significantly less abundant in the fecal microbiota of healthy controls compared with that in patients with HIV, whereas Bacteroidetes was significantly more abundant in the fecal microbiota of healthy controls (P < 0.05, Fig. 2C). At the level of family, Bacteroidaceae was prevalent in healthy controls, whereas the families Prevotellaceae, Sutterellaceae, Erysipelotrichaceae and Enterococcaceae had significantly higher relative abundances in HIV-positive subjects (Fig. 2D). At the level of genus, the abundances of several genera were different in healthy controls compared with those in HIV-positive samples (P < 0.05, Fig. 2E). Among the abundant genera, six (*Bacteroides*, *Dialister*, *Clostridium* XIVa, *Clostridium* XIVb, *Barnesiella* and *Coprococcus*) decreased in abundance in HIV-positive samples, whereas the abundance of ten other genera (*Prevotella*, *Faecalibacterium*, *Phascolarctobacterium*, *Butyricicoccus*, *Erysipelotrichaceae incertae sedis*, *Catenibacterium*, *Dorea*, *Enterobacter*, *Enterococcus* and *Megamonas*) increased significantly in these samples.

Although bacterial diversity was not altered dramatically, the aberrant compositions of fecal microbiota indicated gut dysbiosis in patients with HIV. The ratio of Firmicutes/Bacteroidetes increased significantly in HIV-1-infected samples (1.351 ± 1.761, P < 0.05; Fig. 2F) compared with that in healthy controls in healthy controls (0.482 ± 0.383). Gut microbiota play crucial roles in shaping the local and systemic immune responses during health and disease, and because infection induces chronic gut inflammation, we also evaluated the correlations between fecal microbiota and immune response. Notably, the phylotypes that were enriched in HIV samples were positively correlated with the inflammatory cytokine TNF-α, e.g., *Phascolarctobacterium*, (R = 0.334, P = 0.014; Fig. 2G) and with IL-6, e.g., *Megamonas*, (R = 0.372, P = 0.006; Fig. 2H). For prevalent genera in the healthy controls, *Dialister* was positively correlated with IL-22 (R = 0.620, P = 0.000; Fig. 2I) and IFN-γ (R = 0.287, P = 0.035; Fig. 2J), and *Clostridium* XIVb was negatively correlated with IFN-γ (R = −0.382, P = 0.004; Fig. 2K).

### Effects of HAART on the fecal microbiota in patients with HIV

**Figure 3:**
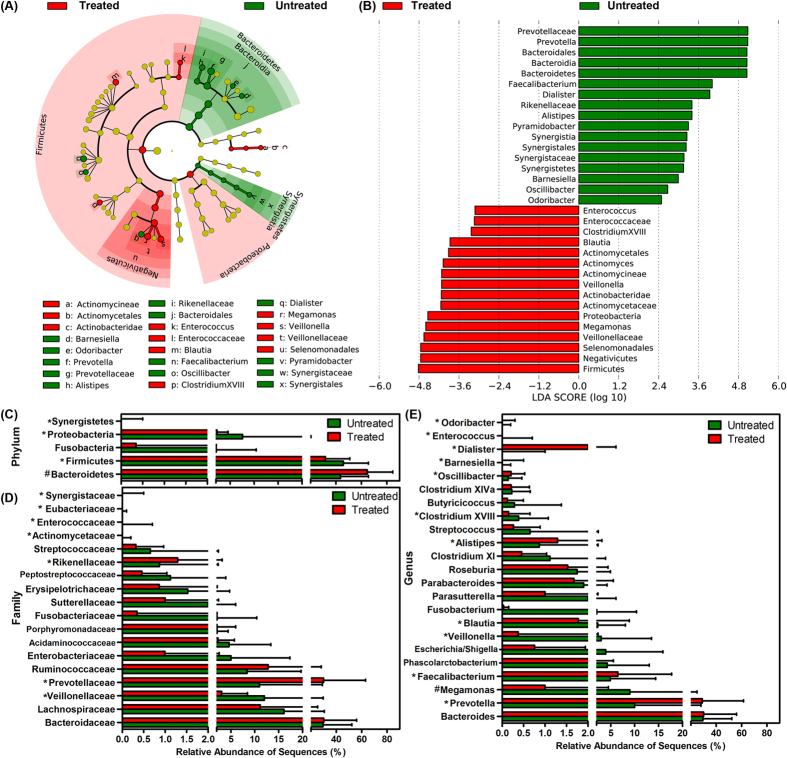
Taxonomic differences between the fecal microbiota of treated and untreated HIV-1-infected subjects. Cladogram represents the features that are discriminative with respect to HAART using the LDA model results for the bacterial hierarchy (**A**). LDA coupled with effect size measurements identified the most differentially abundant taxa between the two groups (**B**). Comparisons of the relative abundance at the level of bacterial phylum (**C**), family (**D**) and genus (**E**) between treated and untreated HIV-1-infected subjects; *P < 0.05; ^#^P < 0.01.

Consistent with our comparisons of fecal microbiota between healthy controls and patients with HIV, no significant differences were detected in the overall microbial diversity between HAART-treated and untreated groups (P > 0.05). However, based on further analysis, HAART might have affected the composition of fecal microbiota in the patients with HIV. The greatest differences in taxa between HAART-treated and HAART-naïve groups, with key phylotypes identified as microbiological markers at different phylogenetic levels, are shown in Fig. 3A,B. Using the LEfSe method, several microbial signatures in the fecal microbiota were different between HAART-treated and HAART-naïve groups. Specifically, Bacteroidetes and Synergistetes were significantly more abundant in the feces of HAART-treated patients than in the feces of those that were HAART-naïve, whereas Firmicutes and Proteobacteria were significantly less abundant in HAART-treated patients (P < 0.05, Fig. 3C). However, the ratio of Firmicutes/Bacteroidetes was not significantly different between the two groups. Of the families, Veillonellaceae, Actinomycetaceae, Enterococcaceae and Eubacteriaceae were prevalent in HAART-naïve patients; whereas Prevotellaceae, Rikenellaceae and Synergistaceae were enriched in HAART-treated patients (P < 0.05, Fig. 3D). Additionally, the genera *Prevotella*, *Faecalibacterium*, *Alistipes*, *Oscillibacter*, *Barnesiella*, *Dialister* and *Odoribacter* were significantly enriched in HAART-treated patients, whereas *Megamonas*, *Veillonella*, *Blautia*, *Clostridium* XVIII and *Enterococcus* sequences were more abundant in HAART-naïve patients. The HAART treatment of HIV patients apparently is effective and reduces the viral load; however, the Firmicutes/Bacteroidetes ratio remained significantly higher than that of healthy controls after HAART treatment (1.751 ± 1.694, P < 0.01). Thus, although it is an effective treatment, the fecal microbiota of the HIV-1-infected patients after HAART treatment were not completely restored. The details are shown in Figure S4 of the differences in fecal microbiota between healthy control and HAART-treated patients. In combination with the previous diversity analyses, our data showed that the composition of the fecal microbiota of the patients infected with HIV-1 was different from that of healthy controls, regardless of HAART treatment. This conclusion may improve the prognosis and lead to the successful treatment of patients suffering from HIV-1 infection.

## Discussion

In this cross-sectional study, we compared the fecal microbiota in HIV-1-infected individuals (untreated and treated with HAART) with that of sex and age-matched uninfected control subjects in a Chinese population using barcoded multiplexed 454-pyrosequencing for the first time. In contrast to traditional microbiological techniques, next-generation high-throughput sequencing techniques provide a relatively comprehensive description of the fecal microbiota associated with HIV-1 infection. This study also provided an interindividual comparison of fecal microbiota to identify the key phylotypes that might be associated with HIV-1 infection. Alterations in the profiles of gut microbiota have been reported in patients with HIV infection in several Western populations^4^,^9^,^13^,^16^,^17^,^18^,^19^,^20^,^21^,^23^,^25^, which find intestinal dysbiosis characterized by enrichment or depletion of specific taxa is associated with HIV infection significantly. As the specific microbiota changes observed during HIV infection are complicated by factors like population, age, sex, duration, sample type, and HAART^29^, the alterations of overall structure of the gut microbiota are not always consistent with each other in previous studies^13^,^16^,^18^,^20^,^23^. Several studies have found that α-diversity in those untreated HIV-infected patients decreased significantly^9^,^17^,^21^, while others have found it decreased obviously after successful HAART^4^,^20^. Consistent with a previous study conducted by Lozupone *et al*.^16^, the present study showed that the overall diversity of fecal microbiota was unaltered in HIV-1-infected individuals^16^, which is significant because the diversity of indigenous intestinal microbiota is one of the key determinants in resisting the colonization of invading pathogens^30^. However, Mutlu *et al*. shows that the lower intestinal mucosal microbiota in HIV-infected subjects are less diverse, definitely distinct from non-HIV controls, and composed more frequently of bacterial populations that are potentially pathogenic^20^. Generally, the mucosal microbiota is less diverse than the fecal microbiota. This decreased level of microbial diversity can make subtle changes in HIV-1 infection much easier to detect or more apparent in mucosal samples, which may be one reason to note a greater difference in the mucosal samples versus the fecal samples^20^. The natural history progression to AIDS from HIV-1 infection is a relatively long-term process^31^, and the unaltered diversity of the fecal microbiota in HIV-1-infected individuals might be associated with the procession and duration of the disease, although it was still difficult to define whether changes in the fecal microbiota were a cause or consequence of HIV-1 infection.

With an average of approximately 8,000 reads per sample, a large number of rare taxa were detected at relatively low abundances, which influence the overall bacterial diversity of the fecal microbiota^32^. However, in recent studies, α-diversity indices, including the number of bacterial species and the Shannon index, were significantly lower in advanced HIV-1-infected patients than those in healthy controls using the Illumina MiSeq platform; this result could be attributed in part to the use of antibiotics and different technologies used to characterize the microbiota^17^,^19^. Antibiotics can cause profound changes in gut microbiota composition that is why so many studies of the gut microbiota have used recent antibiotic usage as an exclusion criteria^19^,^33^,^34^. Our enrolled participants did not use antibiotics in the previous month, and the number of phylotypes in HIV-1-infected patients was obviously higher than that in healthy controls, regardless of whether the group was treated or untreated. Opportunistic pathogens might be the source of the increase in unique phylotypes from the fecal microbiota of HIV-1-infected subjects. Alternatively, the discrepancy in number of phylotypes between studies might be ascribed to a different stage of the disease or to a different ethnic population of the enrolled HIV-1-infected patients. Nowak *et al*. demonstrates that decreased α-diversity measured as the number of bacterial taxa in fecal samples is observed in the highly immune-deficient group (CD4^+^ T cell counts between 120 and 150 cells/μl) compared to other individuals with chronic untreated infection^17^, indicating a potential role for a breakdown in colonization resistance in opportunistic infections that occur with advanced disease. In addition, different next-generation sequencing platforms have different accuracy, coverage rate, and systematic biases, which will influence the following analysis of bacterial diversity and composition^35^. Despite the increased sequencing depth achievable using MiSeq, 454-pyrosequencing (GS FLX Titanium) has been preferably used for 16S rRNA gene-based microbial community analysis, because of its relatively long read length (>450 bp) and high consensus accuracy (99.995%)^36^. In our present study, the α-diversity did not decrease after short-term, effective HAART that suppressed the viral load below the limit of detection. Consistent with previous study, the β-diversity indices such as principal coordinate analysis showed that the fecal microbiota in HIV-1-infected subjects was so different that the patients could be distinctly separated from healthy controls^20^. Therefore, an increase in opportunistic pathogen infections and microbial translocation might be associated with the unaltered overall bacterial diversity that was concomitant with an increase in unique phylotypes in the fecal microbiota of HIV-1-infected participants^18^,^37^. Based on our findings, the short-term HAART inhibited HIV-1 viral replication effectively but did not affect the overall bacterial diversity of gut microbiota. Similar with previous study, our data indicated that the fecal microbiota from HIV-1-infected patients treated with HAART was also clustered with that of untreated individuals infected with HIV-1^16^. Despite successful control of viral load by HAART, HIV-associated dysbiosis of the fecal microbiota was not completely ameliorated. The restoration of fecal microbiota might be highly dependent on the re-establishment of host immunity, whereas short-term HAART did not relieve host immune disorders entirely for those HIV-1-infected patients. In previous studies, dietary supplementation with a prebiotic oligosaccharide mixture or synbiotics resulted in a partial restoration of the composition of the gut microbiota, a reduction in inflammation, the activation of CD4^+^ T-cells (CD25), and an improvement in NK cell activity in HAART-naive HIV-infected individuals^14^,^15^. With current and emerging research providing support for specific benefits of microbiome-targeted therapy in the form of probiotics, prebiotics and synbiotics for those with HIV-1 infection, these dietary supplements could be tolerable, safe and adjuvant therapy for this particular target population.

Although the overall bacterial diversity of the fecal microbiota was unaltered between healthy controls and HIV-1-infected patients in this study, based on statistical analysis, compositional changes in the fecal microbiota were associated with HIV-1 infection. Despite the large interpersonal variations in our study, the relative abundance of Firmicutes increased significantly, whereas that of Bacteroidetes decreased in HIV-1-infected patients, which was inconsistent with previous studies that had largely focused on populations in the U.S. and Europe and not the parts of the world where HIV is the higher prevalent, including China^9^,^18^,^19^,^20^,^22^. In most previous studies in Chinese populations, the relative abundance of Bacteroidetes in the healthy gut microbiota were higher than Firmicutes, whereas Bacteroidetes decreased and Firmicutes increased in the gut microbiota of different diseases^34^,^38^,^39^. This disparity of the baseline microbiota composition might be due to different dietary constitution and host genetic background between Chinese and Western populations. Different dietary patterns have been shown to have significant effects on the microbiota. The Chinese diet was low in fat, sugar, and meat and very high in starch and fiber, which compared with a typical, high-fat, high-calorie “Western” diet. Previous studies have shown that long-term dietary patterns are strongly correlated with the gut microbial enterotypes^40^,^41^,^42^. Individuals on a high-fat diet have a *Bacteroides*-dominated enterotype, whereas a carbohydrate-rich diet is associated with the *Prevotella*-dominated enterotype^40^. These differences in dietary habits between Chinese and Western populations could affect the composition of the gut microbiota and might affect observed changes in the gut microbiota upon HIV-1 infection. Lozupone *et al*.^4^ have demonstrated that diets high in fat and protein and low in carbohydrates and fiber which were consumed by those HIV-infected individuals were associated with the pronounced loss of beneficial bacteria^4^. The ratio of Firmicutes/Bacteroidetes is an important parameter in evaluating the composition of the intestinal microbiota because it may change significantly with the use of antibiotics, specific dietary nutrients and pathological condition. Compared with healthy control, the increased in the Firmicutes/Bacteroidetes ratio in HIV-1-positive patients represented the dramatically changes of the composition of gut microbiota which indicated that dysbiosis of the fecal microbiota was closely associated with HIV-1 infection in the Chinese population. Generally, the ratio of Firmicutes/Bacteroidetes increases from birth to adulthood and is further altered with advanced age^43^,^44^. In previous studies, an increase in the fecal Firmicutes/Bacteroidetes ratio was tightly linked to obesity, irritable bowel syndrome and *Clostridium difficile* infection^45^,^46^,^47^. Effective HAART for HIV infection at least partially improves immune function and eliminates the risk of AIDS-related complications; however, the increase in the ratio of Firmicutes/Bacteroidetes was not reversed in the HIV-1-infected patients treated with short-term HAART, and therefore, the dysbiosis in these patients was sustained^13^. However, Bacteroidetes increased and Firmicutes decreased obviously after short-term, effective HAART. In addition, the proportion of Proteobacteria in HIV-treated individuals decreased into normal level after treatment. Dinh *et al*. demonstrates that the relative abundance of Proteobacteria, specifically of taxa in the Gammaproteobacteria class, including Enterobacteriales and Enterobacteriaceae, is enriched in HAART-treated U.S. cases, whereas the two most dominant phyla such as Firmicutes and Bacteroidetes are not altered significantly^23^. With the same sample type and same sequencing platform as that conducted by Dinh *et al*., the discrepancy might be due to the different surveyed population and dietary constitution^23^. The changing patterns of fecal microbiota after treatment showed a trend to restore the healthy microbiota. The gut microbiota alterations are closely associated with immune dysfunction in HIV-1-infected patients. The importance of microbiota in shaping the development of the gut immune system has been demonstrated experimentally^48^, and the altered fecal microbiota in HIV-1 infection is associated with mucosal and systemic immune activation and endotoxemia^18^. Based on a previous study, dysbiosis is associated with an enrichment of bacterial species that catabolize tryptophan through the kynurenine pathway, which may contribute to the loss of Th17 cells^13^. Previous studies also show that Lactobacillales in anal swabs is negatively correlated with the marker of intestinal microbial translocation such as soluble CD14 (sCD14)^25^, while Enterobacteriales levels in colonic biopsies is positively correlated with it^23^. Although it remained unclear whether altered immunity after HIV infection drives dysbiosis or vice versa, the gut dysbiosis and immune dysfunction were still evident even in the setting of HAART-mediated viral suppression, which might be the treatment dilemma for HIV infection at present.

With the next-generation high-throughput 454-pyrosequencing technique, most of these differential phylotypes could be classified to the level of genus. Consistent with previous studies, we found an increase in the relative abundance of the Gram-negative anaerobe *Prevotella* in conjunction with a decrease in *Bacteroides* in HIV-1-infected patients^13^,^16^,^18^,^20^,^49^. Without sexual behaviors were considered, the *Prevotella*-rich/*Bacteroides*-poor enterotype might be the major characteristics of the HIV-1-associated fecal microbiota in the Chinese population. In a recent study, Noguera-Julian *et al*. reported that *Prevotella* taxa predominated in MSM, whereas *Bacteroides* taxa predominated in non-MSM^21^. However, their conclusions would be challenging, given the need to avoid or minimize confounding by demographics, diet, physical activity, HIV and other infections, and medications particularly antibiotics^50^. As a potentially “pathogenic symbiont” within the microbiota, *Prevotella* are associated with plant-rich diets but are also linked with chronic inflammatory disease states, including periodontal disease and active ulcerative colitis^16^,^51^,^52^. However, the alterations of *Prevotella* in HIV-infected patients is not observed in a small scale cross-sectional study in China, which may be associated with the larger interpersonal variations^53^. In a recent study, the relative abundance of the genus *Prevotella* was positively correlated with the number of activated CD4^+^ and CD8^+^ T cells per gram of mucosal tissue and with the level of CD1c^+^ mDC activation based on CD40 expression^18^. Dillon *et al*. also shows that increased translocation of Gram-negative *Prevotella* into the lamina propria synergizes with HIV to induce intestinal myeloid dendritic cell activation^24^. These results indicate that *Prevotella* participates actively in the modulation of the gut immune system and that the participation changes according to the health status of the host. In this cross-sectional study, we could not determine whether the increased in the relative abundance of *Prevotella* was a cause or a consequence of mucosal immune activation in HIV-1 infection because the relative abundance of *Prevotella* remained higher than that in healthy controls after effective HAART for the HIV-1-infected patients. Koeth *et al*. found that the abundance of *Prevotella* is correlated with higher plasma levels of the pro-atherogenic metabolite trimethylamine-Noxide^54^; however, in our study, the systemic inflammatory cytokines, such as TNF-α, IFN-γ, and IL-6, were not correlated with the abundance of *Prevotella*. We hypothesized that the altered proportions of *Prevotella* might contribute to the local immune response of the GALT but not to the systemic inflammatory response.

In contrast to *Prevotella*, the other differential phylotypes, including the genera *Phascolarctobacterium*, *Megamonas*, *Dialister* and *Clostridium* XIVb, were correlated significantly with the systemic inflammatory cytokines. Notably, the commonly used probiotics such as *Lactobacillus* (belonging to the phylum of Firmicutes) and *Bifidobacterium* (belonging to the phylum of Actinobacteria) were not altered significantly in the HIV-1-infected patients in this study^55^. After short-term, effective HAART, the two genera were not increased obviously. Although the Firmicutes was significantly increased in those HIV-1-infected patients, *Lactobacillus* might be not influence by HIV-1 infection and HAART, which seemed not the major contributor to the increase of Firmicutes. However, in Perez-Santiago *et al*., higher proportions of differential phylotypes at higher taxonomic levels, such as Lactobacillales, are associated with predictive markers of better HIV outcomes that include a higher CD4 percentage, a lower viral load, and less evidence of microbial translocation^25^. Additionally, the proportion of another genus in the phylum of Firmicutes, *Faecalibacterium*, increased significantly in HIV-1-infected patients in our study, which is in contrast to a previous study^17^. As primary producers of butyrate, *Faecalibacterium* have anti-inflammatory properties in addition to being an important energy source in Crohn’s disease patients^56^. In our study, as in that by Vujkovic-Cvijin *et al*., Lozupone *et al*. and Dinh *et al*. reported significant enrichment in the Erysipelotrichaceae family in untreated HIV-1-infected subjects, compared with healthy individuals^13^,^16^,^23^. We also found that the relative abundance of the *Alistipes* genus was slightly decreased in those HIV-1-infected patients. but significantly increased after short-term, effective HAART^13^,^18^,^23^. Consistent with previous studies, another differential genera Barnesiella (belonging to the phylum Bacteroidetes) was decreased obviously in HIV-1-infected patients^18^,^23^, whereas it was increased obviously after successful HAART. Interestingly, four differential abundant genera in the phylum of Firmicutes, the relative abundance of *Phascolarctobacterium*, *Megamonas*, *Clostridium* XIVa and *Clostridium* XIVb were higher in HIV-1-infected patients, but all decreased after treatment. These changes mentioned above were the major contributors to the increased Firmicutes/Bacteroidetes ratio in the HIV-1 infection. All previous studies, including ours, found the association between gut dysbiosis and HIV-1 infection in a Chinese population and identified several biomarkers to represent the gut dysbiosis in these HIV-1-infected patients. The discrepancy between our results and those of previous studies on microbiota might be attributed to differences in the stage of the disease, geography, surveyed population or ethnicity, the sequencing platforms used to characterize the microbiota, and even the computational and statistical methods used to identify differences in gut microbiota composition and differentially abundant taxa.

Our study had several limitations. First, a cross-sectional study, not a longitudinal study, was used to investigate the role of the gut microbiota on the gut immune system, and in a cross-sectional cohort, it cannot be determined whether the altered gut microbiota contributed to or was caused by immune dysfunction. Second, only male HIV-1-infected patients were enrolled in our study, and future studies should include female patients to identify gender disparities. Third, only the effects of short-term HAART were observed in our study and to establish a more meaningful connection between gut microbiota and immune parameters, future studies should investigate the alterations of gut microbiota and the restoration of immune function after long-term effective HAART. Fourth, the microbiota of feces was a proxy for gut microbiota in this study, which was the only realistic sample for a large, noninvasive epidemiologic study. However, fecal microbiota may only represent the composition of the gut microbiota in the lumen and not that on the mucosal surfaces, which is an important distinction because the mucosa-associated microbiota potentially interact with the GALT of HIV-1-infected patients directly. Fifth, sexual behavior of the participants is not controlled in our present study. Controlling for sexual behavior will be crucial moving forward for determining which microbiota differences are related to HIV-induced immune dysfunction versus MSM sexual behavior.

In summary, we demonstrated that individuals with HIV-1 infection, compared with healthy controls, displayed gut dysbiosis characterized by fecal microbiota with unchanged overall bacterial diversity but altered taxonomic composition. Several specific differential phylotypes, i.e., *Phascolarctobacterium*, *Megamonas*, *Dialister* and *Clostridium* XIVb, were significantly correlated with systemic inflammatory cytokines. Although short-term treatment with HAART was effective, the gut microbiota were not completely restored. Future larger-scale, long-term HAART and longitudinal studies that include functional metagenomic and metabolomic approaches to identify the roles of the specific differential phylotypes are required to decipher the relationship between gut microbiota and HIV-1 infection and to provide new insights into the targeted treatment of HIV-1 infection.

## Methods

### Recruitment of subjects

Eighty-three male participants were enrolled in our study from June to July 2014, which included 67 HIV-infected participants from the Departments of Infectious Diseases at the First Affiliated Hospital, School of Medicine, Zhejiang University and Zhejiang Qingchun Hospital, as well as 16 healthy volunteers from the health-screening center. The healthy volunteers were matched with the HIV groups for age, gender, and body mass index (Table 2). All HIV-positive participants were diagnosed and had the disease verified by the Zhejiang Provincial Center for Disease Control and Prevention (PCR and HIV-1 antibody tests). The viral load and peripheral blood CD4^+^ and CD8^+^ cell counts, were determined by standard methods^16^. Cytokine levels in the serum were measured by a quantitative sandwich enzyme-linked immunosorbent assay (ELISA) using a Quantikine kit (R&D Systems, USA). Among the HIV-infected participants, 35 participants were treated with HAART for more than one year; whereas the other 32 were not treated. The following criteria were used to exclude subjects: being female; age >60 y; body mass index (BMI = weight in kilograms divided by the height in meters squared) >30; history of inflammatory bowel disease (IBD); active inflammatory conditions affecting the rectum; opportunistic infection; evidence of hepatitis B or C virus infection; use of antibiotics, probiotics, prebiotics, or synbiotics in the previous month; and use of rectally administered medications, including over-the-counter enemas, within 48 h. The protocols used in this study were approved by the Ethics Committee of the First Affiliated Hospital, School of Medicine, Zhejiang University (China) and were implemented in accordance with the approved guidelines. Informed written consent was obtained from each of the participants before enrollment.

### Fecal sample collection and DNA extraction

When the participants were initially examined at the hospital, approximately 2 g of a fresh fecal sample was collected in a sterile plastic cup and stored in a refrigerator. Samples for bacterial genomic DNA extraction were transferred immediately to the laboratory and stored at −80 °C. DNA was extracted from 300 mg of homogenized feces using a QIAamp^®^ DNA Stool Mini Kit (QIAGEN, Hilden, Germany) according to the manufacturer’s instructions, with additional glass-bead beating steps on a Mini-beadbeater (FastPrep; Thermo Electron Corporation, Boston, MA, USA). The amount of DNA was determined using a NanoDrop ND-1000 spectrophotometer (Thermo Electron Corporation); the integrity and size were checked by 1.0% agarose gel electrophoresis containing 0.5 mg/ml ethidium bromide. All DNA was stored at −20 °C before further analysis.

### PCR and pyrosequencing

The bacterial genomic DNA was amplified with the 27F (5′-AGAGTTTGATCCTGGCTCAG-3′) and 533R (5′-TTACCGCGGCTGCTGGCAC-3′) primers specific for the V1-V3 hypervariable regions of the 16S rRNA gene^33^,^34^,^45^. Each forward primer incorporated FLX Titanium adapters and a sample barcode at the 5′ end of the reverse primer to allow all samples to be included in the single 454 FLX sequencing run. All PCR reactions were performed in 50-μl triplicates and combined after PCR. The products were extracted with the QIAquick Gel Extraction Kit (QIAGEN) and quantified on NanoDrop ND-1000 spectrophotometer, QuantiFluor-ST Fluorometer (Promega, USA) and Agilent 2100 Bioanalyzer (Agilent Technologies, Palo Alto, CA, USA). Equimolar concentrations of 83 samples were pooled and sequenced on a 454 Life Sciences Genome Sequencer FLX system (Roche, Basel, Switzerland) according to the manufacturer’s recommendations.

### Bioinformatics and statistical analysis

Raw pyrosequencing reads obtained from the sequencer were denoised using Titanium PyroNoise software^57^,^58^,^59^. The resulting pyrosequencing reads were filtered according to barcode and primer sequences using a combination of tools from mothur (version 1.25.0; http://www.mothur.org) and custom Perl scripts. Preliminary quality control steps included the removal of sequences shorter than 150 bp with homopolymers longer than 8 nucleotides and average quality score <25, and all reads containing ambiguous base calls or >2 bp incorrect primer sequences. Using mothur implementation of the ChimeraSlayer algorithm^60^, chimera sequences resulting from the PCR amplification were detected and excluded from the denoised sequences. The high-quality sequences were assigned to samples according to the barcodes. The high-quality reads were clustered into operational taxonomic units (OTUs) using mothur^61^. At a 97% level of nucleotide similarity, the OTUs were used for alpha diversity indices (Shannon, Simpson, and Evenness), richness estimators (ACE and Chao1), Good’s coverage, Venn diagram, and rarefaction curve analyses using mothur. A heatmap was generated based on the relative abundance of OTUs using the R statistical software package (version 2.15; The R Project for Statistical Computing, http://www.R-project.org). Phylogenetic beta diversity measures such as the unweighted UniFrac distance metrics analysis were determined using the OTUs for each sample with the mothur program, and principal coordinate analysis (PCoA) was conducted according to the distance matrices created by the mothur and draw 3D graphical outputs using SigmaPlot (Version 12.0, Systat Software Inc., USA)^62^.

Taxonomy-based analyses were performed by classifying each sequence using the Naïve Bayesian Classifier program of the Michigan State University Center for Microbial Ecology Ribosomal Database Project (RDP) database (http://rdp.cme.msu.edu/) with a 50% bootstrap score^63^. The Metastats program through mothur was used to identify the phylotypes that were significantly different among groups^61^. Microbiome features of healthy controls were compared to patients with HIV-1 infection with Metastats using the P-value and the false discovery rate (Q-value) for non-normal distributions. Only taxa with average abundance >1%, P value <0.05, and Q value <0.05 (*i.e.*, low risk of false discovery) were considered significant^64^. The characterization of microorganismal features differentiating the fecal microbiota was performed using the linear discriminant analysis (LDA) effect size (LEfSe) method (http://huttenhower.sph.harvard.edu/lefse/) for biomarker discovery, which emphasizes both statistical significance and biological relevance^65^. With a normalized relative abundance matrix, LEfSe uses the Kruskal-Wallis rank sum test to detect features with significantly different abundances between assigned taxa and performs LDA to estimate the effect size of each feature. A significant alpha at 0.05 and an effect size threshold of 2 were used for all biomarkers discussed in this study.

The correlations between variables were computed using the Spearman Rank correlation. Statistical analyses were performed using the SPSS Data Analysis Program (version 16.0; SPSS Inc., Chicago, IL, USA). All tests for significance were two-sided, and P-values <0.05 were statistically significant.

## Additional Information

**Accession code**: The sequence data from this study are deposited in the GenBank Sequence Read Archive with the accession number SRP066287.

**How to cite this article**: Ling, Z. *et al*. Alterations in the Fecal Microbiota of Patients with HIV-1 Infection: An Observational Study in A Chinese Population. *Sci. Rep.* **6**, 30673; doi: 10.1038/srep30673 (2016).

## Supplementary Material

Supplementary Information

## Figures and Tables

**Table 1:** Comparison of phylotype coverage and diversity estimation of the 16S rRNA gene libraries at 97% similarity from the pyrosequencing analysis.

Group	No. of reads	No. of OTUs^1^	Good’s(%)^2^	Richness estimator				Diversity index	
				ACE	95%CI	Chao 1	95%CI	Shannon	Simpson
Control	119360	2943	98.41%	14008	13387.1–14665.5	7875	7235.1–8610.8	3.916467	0.063107
Treated	287415	4817	98.96%	19732	19057.0–20440.0	11270	10622.5–11990.7	4.303014	0.037468
Untreated	247471	5276	98.69%	20126	19466.3–20816.1	12136	11473.3–12869.8	4.328113	0.042655

^1^The operational taxonomic units (OTUs) were defined at a 97% similarity level.

^2^The coverage percentage (Good’s) and richness estimators (ACE and Chao1) and diversity indices (Shannon and Simpson) were calculated using Good’s method and the mothur program, respectively.

**Table 2:** Descriptive data for subjects in the study.

	Untreated group	Treated group	Control
Number of subjects	32	35	16
Gender	All male	All male	All male
Age (mean ± SD)	36.63 ± 8.05	36.34 ± 8.10	35.7 ± 10.23
BMI(mean ± SD)	22.31 ± 2.70	23.48 ± 2.54	22.83 ± 3.04
Years diagnosed (mean ± SD)	4.34 ± 2.8	4.97 ± 2.18	/
Transmission, no.			
Injection Drug Use	11	4	/
Heterosexual	12	21	/
Homosexual transmission	4	4	/
Drug and sexual	5	6	/
CD4 count (mean ± SD)	352.66 ± 179.63	363.43 ± 185.28	/
Plasma viral load (HIV-1 RNA copies/mL, mean ± SD)	103838.50 ± 157786.55	314.95 ± 289.39	/
HAART years (mean ± SD)	/	2.49 ± 1.42	/
Cytokines			
IFN-γ(pg/mL, mean ± SD)	8.172 ± 4.177	7.377 ± 2.295	10.788 ± 3.852
IL-2(pg/mL, mean ± SD)	0.408 ± 0.894	0.505 ± 1.134	3.310 ± 3.039
IL-6(pg/mL, mean ± SD)	2.576 ± 7.480	19.114 ± 9.725	18.690 ± 10.304
IL-10(pg/mL, mean ± SD)	0.214 ± 0.286	1.187 ± 2.692	1.272 ± 1.523
IL-22(pg/mL, mean ± SD)	0.997 ± 3.890	1.976 ± 3.470	12.524 ± 26.289
TGF-β(pg/mL, mean ± SD)	3653.136 ± 1491.749	2919.154 ± 1035.554	2147.174 ± 1104.937
TNF-α(pg/mL, mean ± SD)	2.164 ± 0.398	10.117 ± 12.494	9.413 ± 9.713

Abbreviations: BMI, body mass index; HAART, highly active antiretroviral therapy; SD, standard deviation.
