# Supplementary material for: Alterations in the Fecal Microbiota of Patients with HIV-1 Infection: An Observational Study in A Chinese Population
Source: Sci Rep. 2016 Aug 1;6:30673. doi: 10.1038/srep30673 (PMC4967929; doi:10.1038/srep30673)
Supplement: Supplementary Information [file srep30673-s1.pdf]

Supplementary Information

Alterations in the Fecal Microbiota of Patients with HIV-1 Infection: An Observational Study in A Chinese Population

Zongxin Ling[1]*, Changzhong Jin[1]*, Tiansheng Xie[1], Yiwen Cheng[1], Lanjuan Li[1§], Nanping Wu[1#]

[1] Collaborative Innovation Center for Diagnosis and Treatment of Infectious Diseases, State Key Laboratory for Diagnosis and Treatment of Infectious Diseases, the First Affiliated Hospital, School of Medicine, Zhejiang University, Hangzhou, Zhejiang, 310003, China

*These authors contribute equally to this work.

[#]Corresponding author; [§]Co-corresponding author

Running title: HIV-1 infection altered fecal microbiota

Corresponding author:

Nanping Wu, State Key Laboratory for Diagnosis and Treatment of Infectious Diseases, the First Affiliated Hospital, School of Medicine, Zhejiang University, Hangzhou, Zhejiang, 310003, China. E-mail: flwnp2013@163.com. Tel.: +86-571-8723-6580; Fax: +86-571-8723-6580.

Lanjuan Li, State Key Laboratory for Diagnosis and Treatment of Infectious Diseases, the First Affiliated Hospital, School of Medicine, Zhejiang University, Hangzhou, Zhejiang, 310003, China. E-mail: ljli@zju.edu.cn. Tel.: +86-571-8723-6458; Fax: +86-571-8723- 6459.

Figure S1 Rarefaction analysis of pyrosequencing tags in the fecal microbiota from HIV-1-infected subjects (n=67) and healthy controls (n=16).

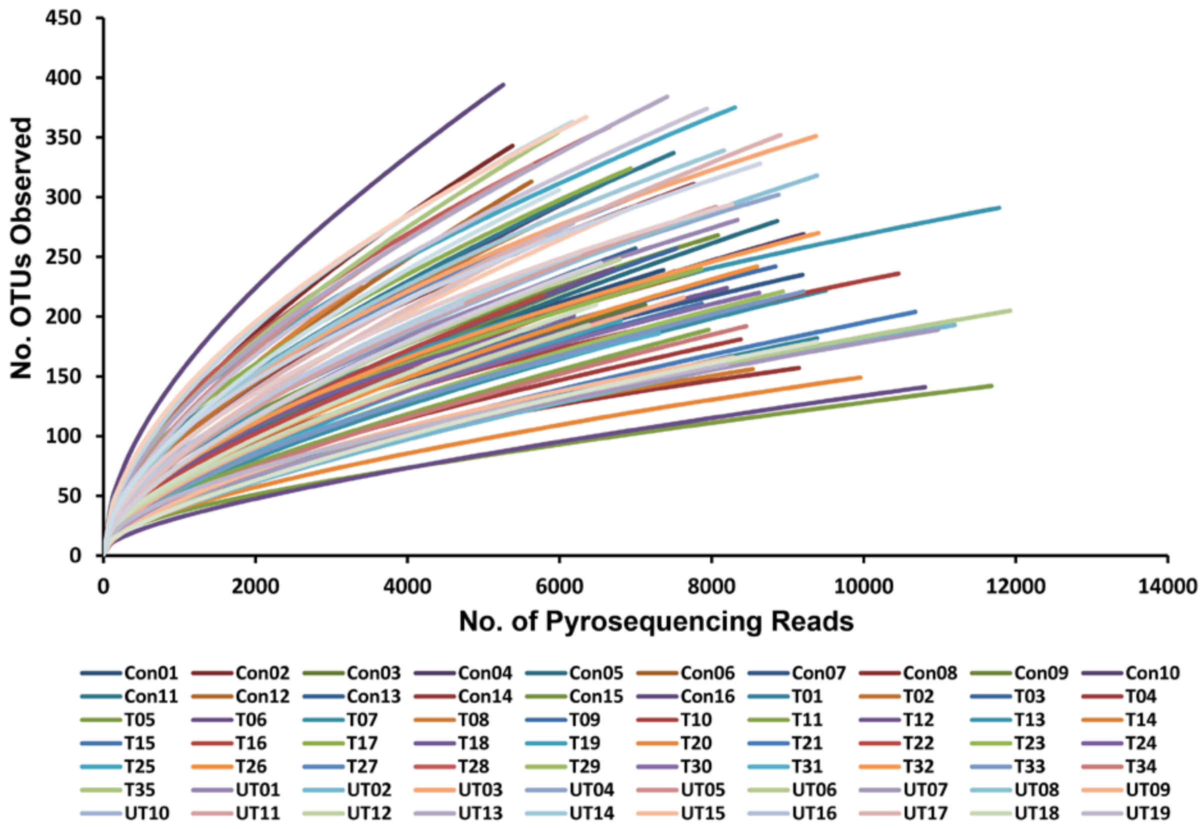

Figure S2 Differentiation in bacterial communities from all participants (interindividual variations). Community differentiation was measured by using the unweighted UniFrac algorithm; the scale bar indicated the distance between clusters in UniFrac units.

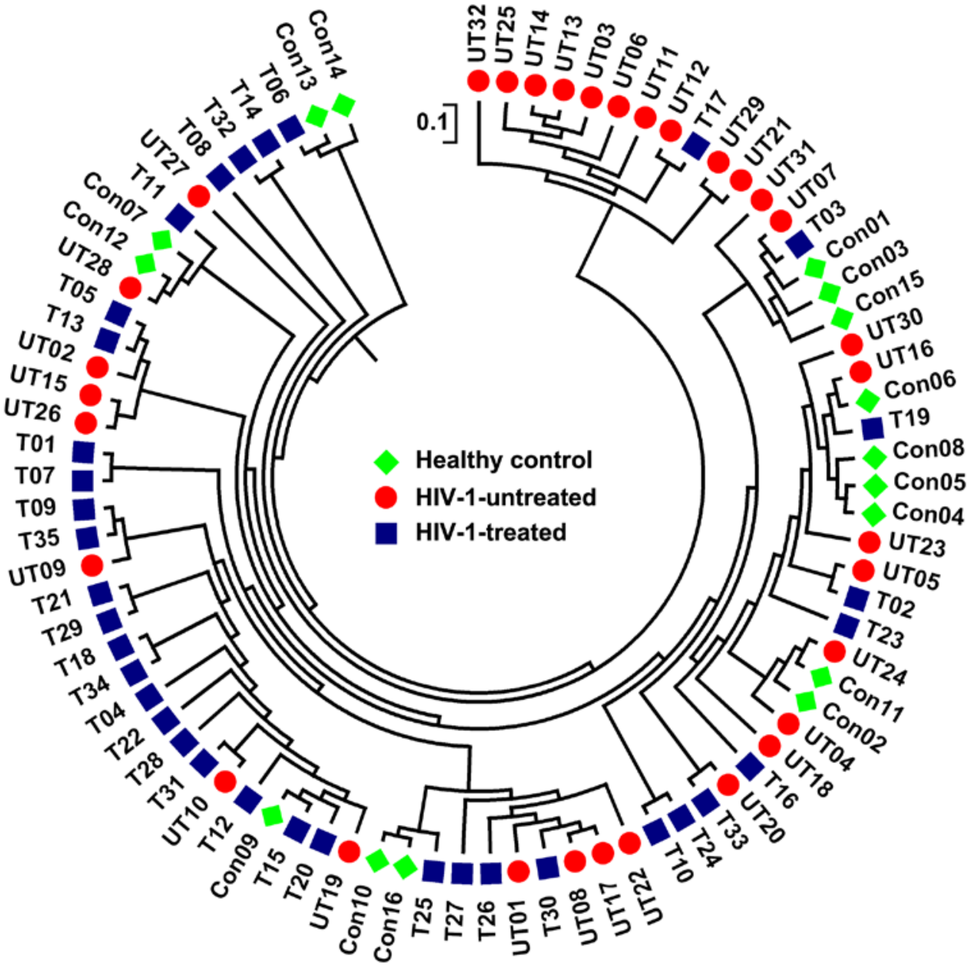

Figure S3 Heatmap indicating the genus-level changes in the healthy control, treated and untreated HIV-1 subjects. The relative abundance of the bacteria in each genus is indicated by a gradient of colour from green (low abundance) to red (high abundance). Complete linkage clustering of the samples was based on the genus-level composition and abundance of the fecal microbiota.

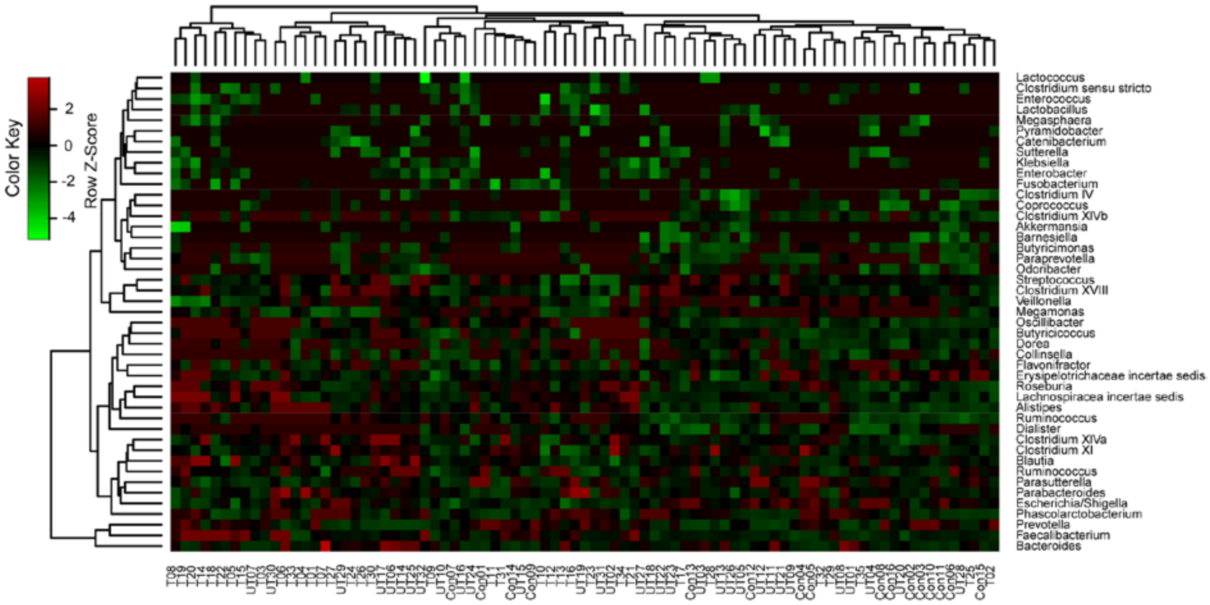

Figure S4 Taxonomic differences of fecal microbiota between healthy controls and HAART treated HIV-1-infected subjects. Cladogram representing the features that are discriminative with respect to treatment using the LDA model results on the bacterial hierarchy (A). LDA coupled with effect size measurements identifies the most differentially abundant taxons between the two groups (B).

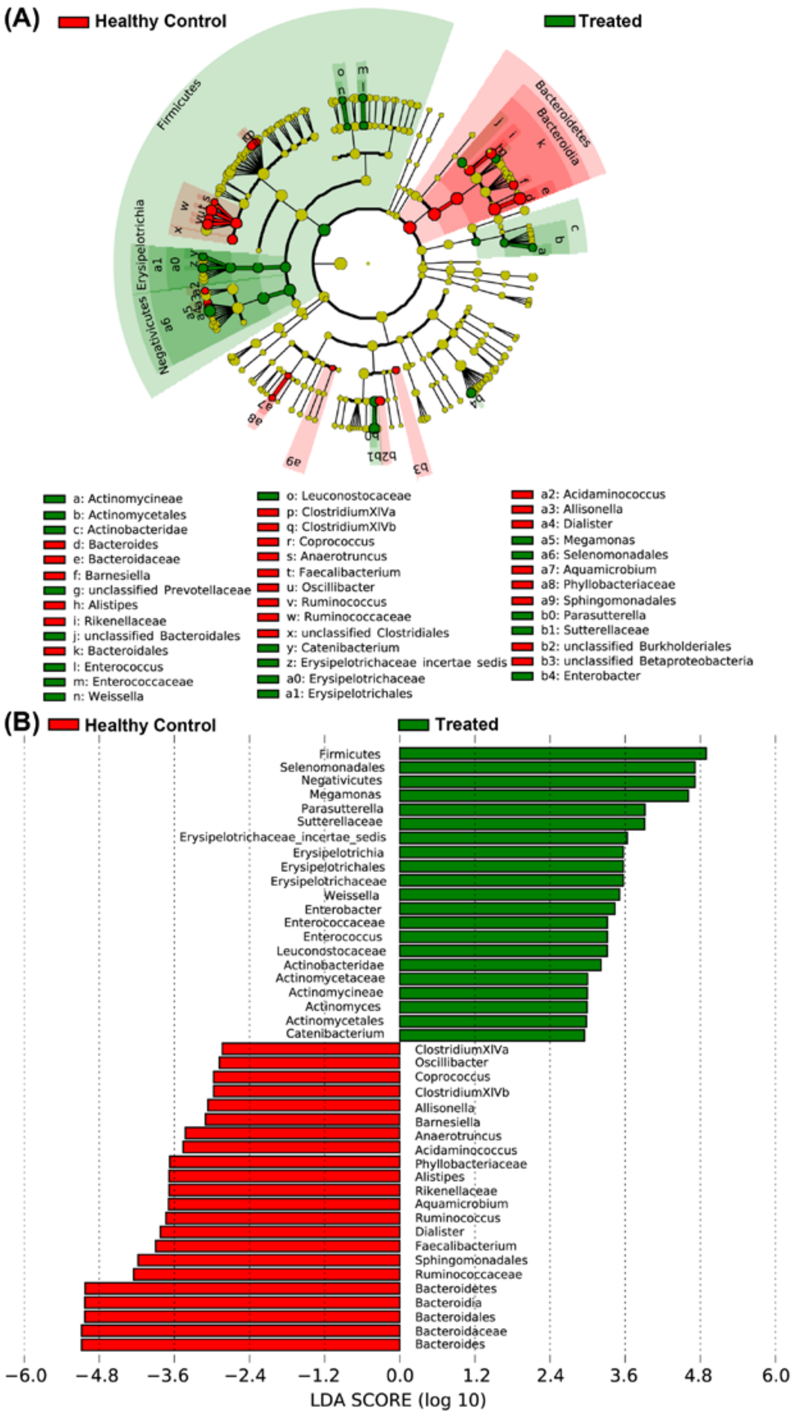

Table S1 Comparison of phylotypes coverage and diversity estimation of the 16S rRNA gene libraries for individuals at 97% similarity from the pyrosequencing analysis

Sample ID	No. of Reads	No. of OTUs	Good's Coverage	ACE	ACE_lci	ACE_hci	Chao1	Chao1_lci	Chao1_hci	Shannon	Simpson
Con01	7365	239	98.0584%	843	714.5	1006.9	630	475.4	883.9	2.085206	0.30297
Con02	5379	343	96.0402%	1387	1216.9	1589.6	953	744.3	1271.0	3.310404	0.086537
Con03	7126	210	98.2599%	821	701.0	970.5	557	412.0	804.7	2.662927	0.141614
Con04	9209	269	98.1974%	1019	879.8	1191.2	684	531.6	924.9	2.006812	0.30957
Con05	8862	280	97.8786%	1691	1467.7	1955.8	813	621.7	1110.4	1.929828	0.346841
Con06	7902	239	98.2663%	914	790.6	1064.6	584	447.7	809.4	1.974576	0.405863
Con07	9194	235	98.5534%	906	780.1	1060.2	549	424.7	753.2	2.51041	0.203632
Con08	7769	311	97.7088%	1251	1098.7	1432.8	677	550.6	871.1	2.592892	0.249259
Con09	7367	254	97.6245%	1771	1533.1	2053.7	798	593.7	1124.3	1.942609	0.358986
Con10	6579	311	96.9144%	1870	1648.7	2128.9	865	673.6	1157.7	2.642677	0.176366
Con11	7498	337	97.2259%	1797	1562.2	2075.7	875	692.9	1151.0	3.499962	0.070839
Con12	5629	313	96.2516%	1346	1140.4	1602.2	965	737.2	1313.9	2.874179	0.166054
Con13	6997	257	97.6847%	1101	951.7	1282.7	678	520.7	928.1	2.109666	0.328161
Con14	9145	157	99.1143%	427	362.4	513.0	328	248.6	474.4	0.98824	0.693169
Con15	8081	268	98.0695%	1133	980.4	1317.7	563	456.9	728.3	2.820013	0.135682
Con16	5258	394	95.4355%	1764	1564.6	1998.0	1238	949.5	1674.9	3.985995	0.04325
T01	9386	182	98.6895%	1321	1109.0	1580.3	523	380.6	767.7	2.006821	0.253362
T02	5767	279	96.9828%	1313	1139.9	1521.5	749	577.2	1020.8	3.063237	0.104074
T03	7534	257	97.7568%	1767	1538.1	2037.3	874	631.7	1273.8	2.545075	0.19649
T04	8381	181	98.6278%	966	811.1	1158.4	567	395.9	872.9	2.010176	0.253947
T05	11678	142	99.2036%	975	781.2	1227.7	427	293.7	678.0	1.795711	0.269719
T06	10807	141	99.1209%	981	808.4	1197.5	404	283.7	624.4	1.264756	0.518475
T07	9502	222	98.4740%	1244	1065.5	1461.3	582	443.1	808.2	2.30018	0.170175
T08	8540	156	98.9813%	546	452.2	668.4	423	295.4	668.3	1.890385	0.337688

T09	8839	242	98.3935%	920	791.5	1078.0	659	491.9	938.2	2.845907	0.116446
T10	10457	236	98.7281%	828	704.2	985.4	561	431.9	775.5	3.121317	0.071766
T11	7959	189	98.3289%	2111	1800.6	2481.9	738	496.9	1166.5	2.31031	0.235507
T12	6703	240	97.5981%	1318	1102.4	1587.4	755	554.6	1083.6	2.770615	0.142851
T13	11782	291	98.7948%	760	670.2	870.3	535	445.4	677.2	2.068306	0.384602
T14	3384	227	95.5674%	1015	852.9	1219.2	641	480.2	903.7	2.965745	0.116657
T15	7870	211	98.3227%	1053	888.7	1256.0	571	424.9	817.6	1.677983	0.486193
T16	5788	218	97.5294%	1012	839.7	1232.2	726	515.0	1085.6	2.572738	0.139605
T17	6935	324	97.2747%	1219	1061.4	1409.2	792	628.5	1041.9	3.231576	0.13887
T18	8200	224	98.4146%	1121	966.4	1307.7	547	417.0	763.0	2.535918	0.165445
T19	6807	197	98.0755%	785	649.1	961.0	567	415.5	824.3	1.723796	0.384495
T20	9954	149	99.0958%	786	653.4	953.2	331	250.2	476.4	1.84007	0.336294
T21	10678	204	98.7076%	1366	1163.0	1612.9	702	491.8	1063.9	2.095248	0.242792
T22	7519	238	97.8854%	1544	1324.5	1808.5	784	567.5	1143.2	2.53784	0.157693
T23	7865	240	98.1691%	1069	913.4	1261.2	621	472.1	866.5	2.845057	0.119943
T24	6186	201	98.2541%	618	524.8	736.9	400	318.3	539.3	2.570513	0.171021
T25	8304	375	97.3748%	1734	1543.3	1955.5	1032	806.7	1374.9	3.758756	0.055153
T26	8597	242	98.2203%	1216	1043.1	1425.5	605	469.0	823.7	2.318289	0.197002
T27	7429	311	97.5905%	1113	975.2	1280.2	754	595.9	998.3	3.295123	0.074561
T28	6657	359	96.9506%	1381	1222.4	1569.7	815	662.7	1042.4	3.579842	0.07813
T29	8937	221	98.3887%	1042	875.6	1251.5	617	461.0	874.5	1.935574	0.285902
T30	8623	220	98.4924%	875	750.3	1028.6	453	363.9	597.1	2.233318	0.265204
T31	7307	186	98.4672%	904	770.4	1068.5	456	342.2	653.5	2.458177	0.149126
T32	9403	270	98.3516%	972	847.6	1123.7	621	491.0	827.7	1.938958	0.41724
T33	9209	221	98.4363%	1255	1059.9	1495.4	650	478.4	935.9	2.182132	0.200203
T34	8453	192	98.4266%	1689	1451.6	1971.3	741	499.9	1169.5	2.013253	0.243916
T35	5975	354	96.2510%	2165	1918.2	2451.2	935	743.6	1219.9	3.477414	0.084811
UT01	8340	281	98.0456%	1111	971.8	1278.5	694	540.7	936.5	2.314617	0.262553

UT02	11197	193	98.9015%	812	666.3	1001.3	427	335.2	579.7	1.988179	0.229832
UT03	9372	351	98.0154%	998	885.3	1133.9	702	584.3	879.4	2.553476	0.262693
UT04	8880	302	98.1306%	925	807.2	1070.2	571	478.3	711.1	2.963957	0.110812
UT05	5643	327	96.4735%	1399	1230.7	1599.7	737	601.5	940.7	2.901321	0.151959
UT06	11927	205	98.8849%	1095	931.1	1295.8	693	483.8	1058.1	1.510835	0.437909
UT07	10976	189	98.9249%	897	754.3	1076.9	518	378.3	759.9	2.166056	0.229721
UT08	9385	318	97.9116%	1516	1330.4	1736.6	796	631.9	1045.1	2.108443	0.371395
UT09	7628	216	98.1384%	1026	850.8	1249.3	601	448.9	852.4	2.302734	0.194369
UT10	6195	247	97.4657%	1176	1006.4	1384.1	892	624.9	1346.4	2.611248	0.191843
UT11	8057	292	97.8032%	1243	1083.1	1435.2	663	533.9	860.5	2.314879	0.252497
UT12	6792	248	97.7326%	1279	1111.0	1480.5	566	449.8	750.3	2.538628	0.19706
UT13	7415	384	96.7633%	2101	1877.9	2356.3	1203	925.4	1624.2	2.842268	0.241447
UT14	8159	339	97.6713%	1093	964.5	1248.3	747	608.5	956.9	2.282657	0.345084
UT15	8286	166	98.8535%	582	490.6	698.1	338	264.0	466.9	1.533477	0.369419
UT16	6175	278	96.8907%	2325	2038.8	2658.3	889	666.7	1239.2	2.299705	0.314296
UT17	8905	352	97.4958%	1752	1548.9	1990.2	1003	783.4	1335.5	2.167558	0.336687
UT18	6383	193	98.0417%	1145	954.3	1384.7	562	407.0	829.3	2.521379	0.147785
UT19	8203	160	98.8419%	717	595.6	872.6	458	318.8	717.8	1.964169	0.290236
UT20	6263	253	97.6369%	997	867.2	1155.5	555	443.1	733.4	2.161628	0.316482
UT21	7020	293	97.2650%	1560	1359.0	1799.5	832	640.8	1129.2	2.745715	0.144268
UT22	4717	211	97.3924%	818	695.6	972.2	479	371.4	658.7	3.03967	0.091952
UT23	5798	265	96.9472%	1471	1267.7	1716.1	752	574.2	1031.2	2.544628	0.159734
UT24	6480	210	97.8704%	1059	872.7	1297.7	574	429.2	813.1	2.600581	0.144595
UT25	7936	374	97.1774%	1478	1316.5	1666.6	1109	854.9	1496.2	2.56309	0.243041
UT26	6167	363	96.6921%	1221	1081.8	1388.1	794	652.3	1006.2	3.445924	0.087257
UT27	6350	367	96.8346%	1366	1218.6	1538.5	769	637.0	965.5	3.573733	0.08645
UT28	8634	328	97.9036%	1119	983.1	1284.1	698	571.1	891.8	3.134086	0.106659
UT29	8268	294	98.1737%	840	738.7	964.3	516	437.8	636.9	2.761314	0.144179

UT30	9386	179	98.7322%	860	708.3	1055.0	484	356.9	702.7	1.973697	0.235656
UT31	6542	245	97.6154%	1009	863.3	1189.3	677	511.4	944.8	2.838396	0.101848
UT32	5992	306	96.8959%	1297	1134.8	1490.0	759	600.3	1002.4	3.154315	0.095472
